# Odor hedonic responses in children and young people with profound intellectual and multiple disabilities

**DOI:** 10.3389/fpsyt.2023.1066286

**Published:** 2023-08-24

**Authors:** Catherine De Blasio, Juliane Dind, Geneviève Petitpierre

**Affiliations:** Department of Special Education, University of Fribourg, Fribourg, Switzerland

**Keywords:** profound intellectual and multiple disabilities, olfactory hedonic processing, olfaction, mood, emotion

## Abstract

**Introduction:**

Odors are closely linked to emotions, play an important role in the well-being of individuals and can influence mood. Despite these crucial properties, the hedonic responses to odors of people with profound intellectual and multiple disabilities (PIMD) remain little explored.

**Aim:**

This within-subjects study aims to examine whether children and young people with PIMD react in a differentiated way to odors evaluated as pleasant or unpleasant by neurotypical adults and, if so, with which behaviors. The influence of their global mood on their emotional responses to odors is also examined.

**Method:**

Twenty children and young people (7–18 years old) with PIMD were exposed to four pairs of hedonically contrasted odors. A control stimulus was presented before each odorant. Five emotional responses, one physiological reaction (nausea reactions), and three responses reflecting approach toward or avoidance of the stimulus were recorded throughout the duration of the stimulus exposure. The participants’ global mood status was measured before the start of the research with the French version of the Mood, Interest and Pleasure Questionnaire (Ross and Oliver, 2003).

**Results:**

The results show that when exposed to pleasant odorants, participants kept their heads aligned with the odorant source longer, smiled longer, and produced more positive vocalizations. In contrast, unpleasant odorants elicit more pouts and grimaces. Nausea reactions occurred in the presence of unpleasant odorants. The hedonic responses were more marked during the second presentation of the stimuli. Participants with a higher MIPQ score showed significantly more emotional reactions to odors.

**Conclusion:**

The results confirm the presence of olfactory preferences in participants with PIMD and the existence of a link between their mood, emotions and olfactory hedonic processing. They prompt the use of odors to support not only the cognitive development of this population, but also their mood and their emotional regulation abilities.

## 1. Introduction

Profound intellectual and multiple disabilities (PIMD) refer to profound cognitive and motor impairments, as well as health problems and/or sensory limitations, mostly due to pre- or peri-natal causes (1, 2). For caregivers concerned about this population’s well-being and mental health, it is important to know how positive or negative affective messages are conveyed by individuals with very limited or non-existent expressive language (3). However, little is known about how people with PIMD express themselves, how differentiated their responses are, and whether their emotions are congruent with their internal states or the characteristics of the stimulus. This study aimed at exploring odor hedonic responses in children and young people with PIMD and to explore the link with their mood.

Odor hedonics refers to the ability to assign valences to perceived odors and express olfactory preferences. Olfactive processing includes detection, habituation, identification, discrimination and preference abilities, that can be studied separately, even if they operate closely together and interact most frequently (4). Experiencing pleasure and displeasure is a fundamental part of life and a core process of motivational and bodily state (5). Odors inducing pleasure are perceived as beneficial by the organism, which tries to approach and/or explore them. In contrast, the organism tries to move away from or reject odors causing displeasure and are perceived as negative. Emotions or feelings of positive value, characterized by enjoyment, happiness or satisfaction, often accompany odors perceived as positive, while emotions of negative value usually induce discomfort or dissatisfaction (6). However, literature in typical population shows that hedonic evaluation differs depending on age, the averages of the pleasantness ratings in children under 12 years old being usually higher than in adolescents or adults (7, 8).

A few exceptions aside, sensory studies in PIMD mainly focused on vision and audition and research on hedonic olfactory processing is still in its infancy (9). However, Vlaskamp and Cuppen-Fonteine (10) found that the reactions of children with PIMD to pleasant and potentially comforting odors were more reliably reported by their teachers than their reactions to other stimuli. An explanation might be that the reactions of children with PIMD to olfactory stimuli are less ambiguous than their reactions to other sensory stimuli, that these children rely more on the sense of smell in comparison to other senses, and/or that the emotional component, which is known to be strongly linked to odor processing, induces more explicit reactions in odorous conditions. Although, Soussignan et al. (11) found that odors elicit spontaneous highly legible reactions in a clinical population made up of children with severe developmental disorders and no verbal language, Lima et al. (12), who presented stimuli from five different sensory modalities (visual, auditory, olfactory, gustatory, and tactile) to three children with PIMD, reported heart rate and electrodermal reactions, but no behavioral responses to two odors (vanilla, orange) and one trigeminal stimulus (vinegar) supposed to induce contrasted emotional reactions. In a second study involving a 3-year-old boy with PIMD, the same authors (13) observed no responses at all for vanilla, physiological responses to orange, and both behavioral and physiological responses to vinegar. They also reported that their participants did not exhibit observable distress or defensive responses to vinegar, a potentially aversive stimulus, but a consistent increase in motor activity, occurring simultaneously with acceleration in heart rate, that nevertheless could suggest some degree of discomfort. The authors concluded that, at least under their exposure conditions in which the stimulus was presented for 5 s only, the behavioral responses of people with PIMD to odorous, respectively trigeminal stimuli, were either limited and hardly visible or absent.

The lack of scientific knowledge about the hedonic olfaction of people with PIMD is problematic because odors are particularly powerful, both in humans and animals. They may relieve pain, reduce stress and promote well-being thanks to the unique characteristics of odor processing and anatomical and psychological ties that it shares with emotions (14–16). First, the chemical senses project onto regions of the brain that also process basic affects and reward, cortical and subcortical cerebral areas involved in the perception of emotion are therefore related to the olfactory structures (17). Second, similarity judgment studies have shown that the affective dimension of smells is one of the most salient, and odors are appropriate stimuli to generate affects (18). Literature reports that they can act on mood (15) as well as on perceived emotions (19–21), the latter being linked to the activation of the autonomous nervous system. Odors can influence the way individuals interact with others and perceive their environment. They can particularly regulate positive or negative emotional states and mood. They also can modulate behavior (toward food for example), cognition, learning processes, memories and reactions to certain events, consciously or not. Finally, beyond its strong emotional tone, olfaction is also characterized by its distal component, enabling a non-intrusive approach (e.g., compared to other emotional distal senses such as gustation or texture sensitivity).

Olfactory experience begins during the third trimester of gestation thanks to the amniotic fluid and the placenta that allow the baby to be in contact with various external odorous substances (e.g., food, smoke, perfume). At the time of birth, life becomes air-dependent and the little human consolidates and diversifies their olfactory experience. Rooted in both prenatal and postnatal experiences, encounters with odors elicit the individual’s various emotions and reactions, including appetence and rejection (22). Most human emotional responses to odors are triggered by association learning and encounters with certain odors in their daily lives play a big role in individuals’ olfactory preferences (14).

Odors elicit behavioral and/or psycho-physiological manifestations, which often occur simultaneously (23, 24) and are observed both in typical children and adults (4). Three types of factors can modulate or alter a response: namely the characteristics of the odorant itself, those of the perceiver, or both of them together (6). The individual’s internal affective state, particularly their mood is a well-known modulator of hedonic response, and vice versa (25). Because they interact closely, overlap heavily and connect in a bidirectional way, emotions and mood are often mistaken for each other (26). However, emotions refer to short-lived, event- or stimulus-driven immediate responses to the anticipation or occurrence of rewarding or punishing stimuli or events, while mood is depicted as a long-lasting affective state which is internally generated and not necessarily induced by a specific situation (27). People with PIMD, children as well as adults, are not spared from low mood or even depression. Families and professionals who describe signs of deep psychological distress in some of them, manifested by agitation, screaming, crying, but also more passive signs, such as the absence of communication, withdrawal, lack of facial expression, attribute their causes to loneliness, regression of abilities, physical pain and/or epilepsy (28). Affective disorders, which are very difficult to assess in this population, who do not communicate verbally (3), are suspected to reach a prevalence of 30% (29). Because they both have limited access to symbolic communication, people with PIMD, like typical young children, rely heavily on their caregivers to modulate their emotions. Self-control is very challenging for them and extremely understudied, even if a few authors however report the presence of simple non-verbal strategies such as, for instance, gaze shifting, behaviorally driven situation modification or response modulation (30, 31).

Considering the need to maximize the well-being of people with PIMD and to promote support to them, and in light of the identified gaps in the current knowledge about how individuals with PIMD process pleasant or unpleasant odors and the fact that, in the general population, the hedonic processing of odors is related to mood, this study raised the three following research questions: (1) What kind of behavioral manifestations do children and young adults with PIMD express when presented with a pleasant vs. an unpleasant odorant? (2) Are these behavioral manifestations hedonically differentiated? (3) Does the global mood of children and young adults with PIMD influence their behavioral responses to odors? Do age (puberty), or feeding mode, also influence these responses?

## 2. Participants, materials and methods

### 2.1. Ethics and general design

Three specialized schools participated in the study. Ethical approval was given by the Geneva and Vaud Ethics Committees (ID: 2019-00234). Written consent was obtained from parents or legal guardians before the start of the study. To respect the well-being of the highly vulnerable participants, the session was postponed if the participant’s state of alertness, fatigue or health required it. Participant’s participation was permanently discontinued if they showed discomfort after two sessions.

A within-subjects experimental design was chosen to explore the research questions. The experimental manipulation of the odors appeared to be the better option to study odor processing in this population. Previous literature has indeed noticed that smelling behaviors are difficult to observe in ecological conditions and that, in such contexts, the researchers themselves are not always aware of the presence of the odors (9). We decided against creating a control group. The first reason is the insoluble problem of finding matching criteria with people with PIMD (32); the second lies in the limited constructive scope of comparative studies that focus on the difficulties of people with disabilities compared to typical people, and not on the functioning of people with disabilities *per se*.

### 2.2. Participants

#### 2.2.1. Selection criteria

Direct support workers from the three participating schools were asked to select all participants meeting the following inclusion criteria: (1) presenting the key characteristics of PIMD (1); (2) being aged between 7 and 18 years and (3) attending the school for at least 6 months at the time of recruitment. Children and young people presenting food allergies or chronic airway problems with indication of daily respiratory therapy were excluded.

#### 2.2.2. Experimental sample—demographic characteristics

Thirty-one children and young people were identified. However, four were removed from the list after a thorough check between the researchers and the schools due to uncertain key characteristics of PIMD; two families declined the participation call; three participants left the study at the very beginning of the data collection: two because of severe health problems and a third because he could not stand the experimental setting. A further two left due to organizational constraints at the time of data collection, including a hospitalization. The final sample contained 20 participants aged between 7 and 18 years (*M* = 13.2 years; SD = 3.5), comprising two 7-year-olds, one 8-year-old, two 10-year-olds, two 11-year-olds, one 12-year-old, two 13-year-olds, two 14-year-olds, one 15-year-old, two 16-year-olds, three 17-year-olds and two 18-year-olds*. Clinical characteristics*: Most participants were impacted by pre- or perinatal causes (i.e., maternal hemorrhage during pregnancy; neonatal anoxia; epileptic encephalopathy of genetic origin; neonatal herpes simplex virus infection; adrenoleukodystrophy; Christianson syndrome; Wolf-Hirschhorn syndrome; startle syndrome; partial trisomy 9; cerebral palsy; multicystic encephalomalacia). PIMD was acquired in two participants, one as a result of an acute encephalopathy when he was 2 years old, the other because of a head injury at 18 months. Etiology was unknown for two and the onset of epileptic encephalopathies uncertain for the last two (Table 1).

**Table 1:** Participants’ characteristics.

Demographics	(%)
**Gender**	
Male	14 (70%)
Female	6 (30%)
**Etiology**	
Known or strongly suspected within	18 (90%)
Pre- and perinatal^a^	14 (70%)
Postnatal	2 (10%)
Time of onset not available	2 (10%)
Unknown	2 (10%)
**Epilepsy**	
Seizures controlled by medication	9 (45%)
Seizures once a month	2 (10%)
Seizures once a week	3 (15%)
Seizures once a day	1 (5%)
No epilepsy	5 (25%)
**Visual impairment**	
Confirmed	16 (80%)
No visual impairment	4 (20%)
**Auditive impairment**	
Confirmed	1 (5%)
Presumed	2 (10%)
No auditive impairment	16 (80%)
Missing	1 (5%)
**Tactile impairment**	
Confirmed	5 (25%)
Presumed	6 (30%)
No tactile impairment	8 (40%)
Missing	1 (5%)
**Medication**	
Medicated	19 (95%)
Non medicated	1 (5%)
**Feeding mode**	
Blended food	7 (35%)
Chunky food	3 (15%)
Enteral feeding (partial)	9 (45%)
Enteral feeding (complete)	1 (5%)

^a^Onset through seven days after birth.

### 2.3. Odor stimuli

#### 2.3.1. Selection of odorants

A set of 18 stimuli was preselected by the researchers in collaboration with one of the schools that initiated the research. Odors that were common or likely to be encountered by participants in their daily life were given priority. The hedonicity of the stimuli was evaluated by neurotypical adults and not by the participants on a 3-level categorical scale: U=Unpleasant; N=Neither pleasant nor unpleasant; P=Pleasant. The adult group comprised 8 men and 21 women: three 18–29 years old, twelve 30–39 years old, eight 40–49 years old, four 50–59 years old and one 60–69 years old. Odor perceived intensity was determined by the same individuals on a 9-level Likert scale (1 = no odor at all to 9 = extremely strong odor). The concentration of non-commercial odorants was manipulated to select those of medium intensity according to neurotypical judgments (33, 34). Eight hedonically contrasted odorants were selected. Two odors came from the standardized “Sniffin’ Sticks Test” manufactured by Burghart Messtechnik GmbH,^1^ five were produced by the Swiss manufacturer of flavors and fragrances Givaudan, Geneva, one, hand sanitizer [Sterilium^®^], comes from a commercially available product and was chosen because it was used by school staff for their own hand hygiene. An odorless stimulus—dipropylene glycol—was used as control.

#### 2.3.2. Stimulus properties

Four of the eight stimuli were considered pleasant by typical adult raters, and four unpleasant. Intensity ranged between 3.59 and 6.45, with an average of 5.01 for pleasant and 5.59 for unpleasant stimuli on a scale of 9. Participants’ stimuli familiarity, checked by their parents or legal guardians on a binary scale (0 = unfamiliar or not familiar at all; 1 = somewhat familiar or very familiar), ranged between 0.13 (lily of the valley) and 0.81 (cheese), with an average of 0.34 for pleasant and 0.51 for unpleasant stimuli on a scale of 1.

#### 2.3.3. Stimulus delivery device

**Figure 1:**
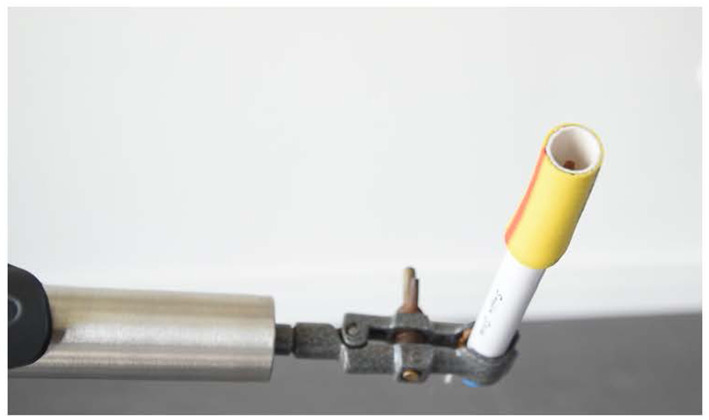
Clip holding the delivery device with its protective cylindrical ring.

The odors were presented at ambient temperature in a 14 cm long and 1.3 cm diameter Burghart pen-like device (Burghart Medical Technology) using a tripod with an articulated arm and a clip. The device was made from an adjustable microphone stand with a telescopic 70 cm long swivel arm to prevent the researcher from bringing his or her hand too close to the participant’s face and hiding their face. During the odor presentation, the tip of the stick was protected by a rubber tube to prevent direct contact with the mouth or any other part of the participants’ face (Figure 1). Each stick was stored in a hermetically sealed glass container to avoid contamination and to minimize loss due to solvent evaporation between sessions.

#### 2.3.4. Stimuli delivery sequences

Stimuli were combined to form food and non-food pairs, respectively. The first, which are usually processed retronasally, may elicit a different hedonic experience in comparison to the second, which have a high orthonasal but low retronasal familiarity (35). Food pairs were: orange-garlic and cinnamon-cheese. Non-food were: lily of the valley-sweat and summer rain-hand sanitizer odors. Each pair teamed a pleasant (O_P_) with an unpleasant stimulus (O_U_). See Table 2 for the characteristics of the pairs.

**Table 2:** Identity, concentration, origin and average values (±standard deviations) of odor properties as reported by a panel of neurotypical adults.

	Pair 1		Pair 2		Pair 3		Pair 4	
	Orange (Burghart)	Garlic (Burghart)	Cinnamon	Cheese	Lily of the valley	Sweat	Summer rain	Hand sanitizer
Hedonicity	Pleasant	Unpleasant	Pleasant	Unpleasant	Pleasant	Unpleasant	Pleasant	Unpleasant
**Intensity**								
*M*	5.00	6.45	6.10	5.07	5.36	5.66	3.59	5.21
SD	(0.92)	(0.91)	(0.93)	(1.33)	(1.02)	(1.44)	(0.93)	(1.17)
**Familiarity**								
*M*	0.48	0.38	0.29	0.81	0.13	0.33	0.48	0.52
SD	(0.48)	(0.49)	(0.46)	(0.40)	(0.35)	(0.48)	(0.51)	(0.51)
**Concentration**								
%	NA	NA	10	1	20	1	50	NA

NA, Non available.

### 2.4. Experimental setting

#### 2.4.1. Physical environment

The experiment took place in the participants’ schools. Quiet, easily ventilated rooms, far from the kitchen and/or places that may generate odors (laundry), were used. To limit competing visual or auditory stimuli during the sessions, adjustments were made such as drawing the curtains, partially lowering the blinds, hiding potentially attractive objects, etc. The experimenters and the teacher were careful not to wear perfume and not to bring food into the rooms. The researcher presenting the stimuli wore cotton gloves to prevent contamination of the odorants when handling the sticks. The rooms were aired before and after each session, as well as before and after each participant. A white 12.5 cm x 12.5 cm grid was used to harmonize the flat surface behind the participant and provide marks for adjusting the zoom. It also facilitated the coding of some indicators, such as head displacements. The rest of the material consisted of a transparent pouch containing numbered cards corresponding to the steps of the study and the odorants presented. This pouch was fixed on the black screen behind the participant and the experimenter changed the cards as the stimuli were presented. Finally, the equipment included a poster informing that the experiment was in progress to avoid inopportune interruptions, photographs of the participant and experimenters, as well as pictograms to present the stages of the session to the participant and a small bell to announce the beginning and end of the test to the participant.

#### 2.4.2. Participants’ condition

The test procedure took place during school time (9–11:30 or 13:45–16:00). Sessions and lunchtime were a minimum half hour apart to limit the influence of the participants’ satiety or hunger state on their hedonic olfactory responsiveness. The interval was respected independently of the participants’ feeding mode (blended, chunky, partial or full enteral), the influence of which on hedonic responses is unknown. As epilepsy is a high comorbidity in people with PIMD and can cause loss of consciousness and altered perception of the environment, monitoring it throughout the session was of great importance. The presence of a direct support worker who knew the participant well was systematically organized during the data collection to alert the researchers to possible seizures and ensure the participant’s well-being and safety. Before each session, the researchers asked about the participant’s current state of health and alertness and decided with the teacher if the session could take place or would have to be postponed. Finally, determining the positioning of the participant during the presentation of the odors was essential so that, to the extent of their possibilities, the participant had as much freedom of movement as possible. For this reason, a member of the research team met every participant a few weeks before the data collection and asked about the type of personal seat used by the participant to be sure that this seat allows enough freedom of movement and minimizes tonic and motor fluctuations. If necessary, a better postural adaptation was sought with the participant’s physio- and/or occupational therapists, i.e., introduction of a headrest or support behind the neck for the sessions. The pilot study confirmed that the sitting position in the participant’s own seat was favorable to present the odors.

#### 2.4.3. Social environment

The experiment was attended by the participant, a direct support worker who knew the participant well, and two researchers. At the time the odors were presented, the experimenters and the teacher refrained from speaking to the participant so that they could focus their full attention on the olfactory stimuli. Nonverbal interactions with the participant were also kept to a minimum (without appearing totally indifferent) so as not to bias the observations.

### 2.5. Experimental procedure

#### 2.5.1. Experimental design

To avoid a possible fatigue and olfactory saturation effect, data were collected in four sessions –one pair of odors per session– spaced about 1 week apart (one session/week for 4 weeks). The total assessment time for each session was 4 min per participant. Of the 20 participants, 19 completed the four sessions, i.e., were exposed to the eight odors, while one was only exposed to six odors as he was absent at a session. The dataset results in a total of 158 records (19 × 8 + 1 × 6). Each session involved the odor pairs and the control stimulus being presented twice (T1 and T2) alternately for 30 s each. Thirty-second exposure was chosen to be enough long to observe how the participants react and cope emotionally with the odor. For the interstimulus interval, 30 s are usually considered enough time for the olfactory recovery between two odorous stimuli (36). We decided to not counterbalance the order of the stimuli across the sessions to (1) avoid exposing participants to two successive unpleasant stimuli at the start or end of the session and (2) because in contrast to typical participants, participants with severe neurodevelopmental disorders do not exert control over their spontaneous facial configuration (11). Their responses are therefore unlikely to be biased by social expectations. Table 3 shows the sequence of stimuli presentation for each pair.

**Table 3:** Sequence of presentation of pairs of odors.

	T1				T2			
Stimulus	Control stimulus	Pleasant odorant	Control stimulus	Unpleasant odorant	Control stimulus	Pleasant odorant	Control stimulus	Unpleasant odorant
Duration in seconds	30	30	30	30	30	30	30	30
Time flow in seconds	0–30	30–60	60–90	90–120	120–150	150–180	180–210	210–240

#### 2.5.2. Procedure

The experiment was preceded by (1) a pilot study and (2) a preliminary contact with the participant. The pilot study was conducted with five children and young adults with PIMD who were not part of the sample. The aim was to decide how the stimuli should be presented (sticks or bottle), to test the procedure (session duration, tolerance of participants toward the setting, experimenters’ and participants’ locations) and to confirm the predicted indicators. The pilot study showed the advantage of sticks over bottles in terms of ease of handling. It also showed the need to add the protective rubber tube at the end of the stick. It confirmed that the participants were tolerant toward the setting and the duration of the sessions. Finally, it confirmed the selection of the panel of indicators.

The purpose of the preliminary meeting was to allow the experimenter to meet each participant for half a day, around 2 weeks before the experiment, to get to know them and discuss adaptations and organizational points with their teachers (i.e., when, morning or afternoon, the participant’s state of awareness was more favorable, determining the positioning of the participant during the presentation etc.).

#### 2.5.3. Experimenters’ operational roles

Two researchers and the participant’s teacher or educator were present during the data collection. The researcher, who presented the stimuli to the participant, stood either to the right or left of the participant, alternating from one session to another to avoid participant orientation bias. The second researcher oversaw time indications and made sure that both cameras were working properly. The participant’s teacher or educator attended the session for the safety reasons described above. The researcher who presented the stimuli to the participant opened the stick, fixed it on the clip and presented it about 2 cm in front of the participant’s nose, between their nose and chin (12). Sessions were videotaped by 2 digital camcorders located 2 meters in front of the participant, one focusing on the participant’s face, the other on his or her body. For close-up framing, the camera focus on the participant’s face was 3 background black screen squares high by 5 wide (37.5 × 62.5 cm). For wide frame, the participant had to be seen from head to toe. Neither the researchers nor the teacher or educator spoke to the participant between the beginning and the end of the presentation of the odorants. The researchers were not blind to the kind of stimulus presented.

### 2.6. Behavioral variables and coding

#### 2.6.1. Definition of the variables

Five hedonic indicators, i.e., smiles, positive vocalizations, pouts, negative vocalizations, emotional outburst, plus nausea reactions as physical response, were recorded throughout the duration of exposure to the stimuli. The selection of these indicators was based on two rationales: (1) that they have been reported in the developmental literature [(37) for a systematic review] and that they (2) belonged to the register of behaviors of people with PIMD (3, 9, 38, 39). As a positive hedonic attribution usually induces approach or avoidance behavior (5), three more indicators were used, namely: head alignment on the stick, nose/hand coordination on the stick and attempts to reject the stick, resulting in nine indicators in total. Depending on the relevance for the target behavior, five behaviors were coded on duration (head alignment on the stick, nose/hand/stick coordination, smile, positive vocalizations, negative vocalizations) and four on occurrences (pouts, attempts to reject the odor, emotional outbursts and nausea reactions). This choice was validated by the practicality of the measurement, methodological recommendations (40), coding procedure used in olfaction research on newborns and young children (41), and the results of the pilot study.

#### 2.6.2. Approach or avoidance responses

The head alignment on the stick was coded when the participant was holding their head up (immobile or with very slight movements) and aligned on the axis of the stick (the projected straight line as a continuation of the stick reaching the nostrils). With regard to the stick rejection, the behavior had to appear within 2 s after the insertion of the stick, the participant pushing either the holder or the stick away from under their nose or genuinely turning their head away while it was aligned with the odorant at the moment of presentation, i.e., the angle of the head in relation to the stick ends up at about 45° (perpendicular). Nose/hand coordination on the stick was coded when the participant grasped the stick under their nose without moving it maintaining the nose motionless aligned with the stick, or making small lateral head movements the nose on the stick or when they applied lateral or vertical movements to the stick in the proximity of their nose.

#### 2.6.3. Hedonic responses

Smiling was defined as the elevation of the two corners of the mouth (42), with the mouth open or closed (43); rictus due to epileptic manifestations or paradoxical smiles and social smiles directed toward persons were excluded. Pouting referred to momentary deformations of the face. i.e., frowning the nose to express displeasure (pouting) or disgust (grimacing). Positive vocalizations referred to sounds produced with the vocal cords in a “positive” (e.g., cooing with pleasure, babbling) mode (44), or to vocal sounds made while smiling. Plaintive vocalizations, such as whining, moaning, screaming and/or irritated/aggrieved/enervated vocal sounds, were defined as negative vocalizations. The sounds could be single, regardless of their duration, or linked, i.e., a series of close vocal sounds, each one less than 1 s apart from the one preceding or following it. Emotional outburst refers to massive emotional reaction (e.g., cries of distress, crying, trembling, paradoxical laughter).

#### 2.6.4. Physiological response

Nausea reactions refer to rapid and intense contraction of the pharynx, causing the opening of the mouth and usually the protrusion of the tongue (heave).

#### 2.6.5. Coding

The coding was carried out by a team of three coders experienced in video analysis, two of them with the target group. Two had a doctoral degree in special education, the third a MA degree. Only one coder was fully blind to the odor being tested. The coding was done both in continuous and real-time measurement. The direct support workers were consulted when the coders had a doubt about certain behaviors. The coding procedure was set up in the EUDICO Linguistic Annotator (ELAN) Software version 5.9 (Max Planck Institute for Psycholinguistics, Nijmegen, The Netherlands). A refined reliability assessment procedure using EasyDIAg algorithm (45) was used to check both the observers’ agreement about presence/absence of a behavior and the temporal overlap of their coding. The reliability value (kappa) was calculated on 20% of the data. The accuracy of the grid makes it possible to reach a satisfactory interobserver agreement for 5 out 9 indicators: head alignment on the stick (*k* = 0.83); smiling (*k* = 0.72); pouting (*k* = 0.77); rejecting the stick (*k* = 0.85); positive vocalizations (*k* = 0.80). The interjudge of four indicators could not be calculated due to too few data. Full details on the coding procedure are available in a previous publication (33).

### 2.7. Measurement of mood

Nonverbal communication prevents people with PIMD from self-reporting on their emotional state (46, 47). The Mood, Interest and Pleasure Questionnaire (MIPQ) (48) was used to identify the participants’ affects. This 23-item Likert hetero-reported questionnaire is based on definitions of low mood and anhedonia outlined under the criteria for major depressive episode in DSM-IV (49), as well as on studies validating behavioral indices of the measured constructs in the population with PIMD (50). The total score is a maximum of 92 points, high scores denoting high mood and interest and pleasure levels. Both the internal consistency and interrater reliability of the French version are good [Cronbach’s *alpha* = 0.97 for the total scale; Pearson *r* = 0.90, (51)]. The study required the participant’s teacher or a member of the educational team to complete the MIPQ with reference to the two preceding weeks.

### 2.8. Statistical analyses

As the data was not normally distributed, nonparametric statistics were used to perform the analyses. A Wilcoxon signed ranks test was used to compare the responses between pleasant and unpleasant modalities as well as at time 1 and time 2. Effect sizes for the Wilcoxon signed ranks test were calculated according to Pallant (52) that is by dividing the *Z* value of the test by the square root of the number of observations [*r* = Z/√(n1 + n2)]. Confirmatory analyses comparing control, pleasant and unpleasant modalities, were performed with Friedman tests (χ^2^), alpha level at 0.05 (unilateral) with Dunn-Bonferroni *post hoc* tests with a correction for multiple testing being used for pairwise comparisons. Effect sizes were carried out with Kendall *W* statistics. The role of the mood, age (puberty), and feeding mode, on the olfactory responses was investigated thanks to generalized linear mixed models (t). The participants were divided into two groups based on their MIPQ scores for this analysis. Group 1 includes participants who scored less than 60 in total and/or obtained low scores on 2 out of the 3 MIPQ subscales (i.e., positive mood score ≤ 23, interest score ≤ 17 or negative mood score ≤ 17) and were therefore considered to have a low general mood. Group 2 includes participants who scored above these cutoffs. Two groups were also formed to study the influence of puberty (“up to 11 years” vs. “12 years old and over”), and two others to study feeding mode (“blendy and chunky” vs. “complete or partial enteral feeding mode”). All analyses were performed using SPSS software version 26.0.0 (IBM Corp.). No analysis was carried out for the indicator “emotional outburst, which did not obtain any occurrences.

## 3. Results

### 3.1. Participants’ responses to pleasant vs. unpleasant odors and control stick

The first analysis aimed to answer the first two research questions, namely, to explore if the participants exhibited differentiated behaviors in the presence of pleasant vs. unpleasant odors and, if so, how? For this analysis, the participants’ (*n* = 20) responses to all pleasant odorants (orange, cinnamon, lily of the valley, summer rain) were compared to those to all unpleasant odorants (garlic, sweat, cheese, hand sanitizer) on time 1 (T1) and 2 (T2) aggregated (Table 4). For greater clarity of the results, we replaced the medians (Mdn), which were often small, by the means (*M*) and the standard deviations (*SD*) in the Wilcoxon statistics. The results show that the participants responded significantly differently when presented with pleasant vs. unpleasant odors for five of the nine responses studied. They emitted more positive vocalizations (*M*_Pleasant_ = 1.09 s, SD = 3.51, *M*_Unpleasant_ = 0.36 s, SD = 1.56, *Z* = −3.05, *p* = 0.001, *r* = 0.25) and smiled longer when presented with pleasant odors than with unpleasant odors (*M*_Pleasant_ = 4.11 s, SD = 7.75, *M*_Unpleasant_ = 2.81 s, SD = 6.08, *Z* = −1.77, *p* = 0.039, *r* = 0.14). Faced with unpleasant odors, the participants exhibited significantly more pouting in comparison to pleasant odors (*M*_Unpleasant_ = 1.78, SD = 2.27, *M*_Pleasant_ = 0.70, SD = 1.82, *Z* = −4.60, *p* < 0.001, *r* = 0.37). It was also noticed that nausea reactions were manifested only in the presence of unpleasant stimuli (*M*_Unpleasant_ = 0.12, SD = 0.54, *M*_Pleasant_ = 0, SD = 0, *Z* = −2.06, *p* = 0.020). Because of the scarcity of the nausea reaction manifested by four participants with three unpleasant odors out of four, i.e., garlic, sweat and hand sanitizer triggered such a response, but not cheese, the effect size was small (*r* = 0.17). Finally, about approach or avoidance behaviors, participants aligned their heads in line with the source significantly longer when they were exposed to the pleasant odors compared to the unpleasant (*M*_Pleasant_ = 27.24 s, SD = 15.76, *M*_Unpleasant_ = 24.32 s, SD = 15.18, *Z* = − 2.11, *p* = 0.017, *r* = 0.17).

**Table 4:** Responses per odor (times 1 and 2 aggregated).

Responses (in seconds)	Orange *N* = 20		Lily of the valley *N* = 20		Summer rain *N* = 19		Cinnamon *N* = 20		Garlic *N* = 20		Sweat *N* = 20		Hand sanitizer *N* = 19		Cheese *N* = 20	
	*M*	SD	*M*	SD	*M*	SD	*M*	SD	*M*	SD	*M*	SD	*M*	SD	*M*	SD
**Positive**																
Smiling	2.93	5.60	5.44	7.78	3.75	6.72	5.17	10.33	2.36	6.41	2.01	5.37	4.00	6.19	3.33	6.24
Positive vocal.	0.52	1.00	0.56	1.16	1.37	3.46	2.08	5.79	0.21	0.47	0.20	0.63	0.36	0.85	0.84	2.88
**Negative**																
Pouting	0.70	1.38	1.25	3.04	0.11	0.32	0.60	1.10	2.10	2.57	1.70	2.66	1.37	1.80	1.65	1.93
Negative vocal.	0.37	1.67	0.16	0.55	0.09	0.39	1.13	4.12	0.14	0.35	0.17	0.42	0.20	0.77	0.72	2.00
**Physiol. react.**																
Nausea react.	0	0	0	0	0	0	0	0	0.25	0.91	0.15	0.49	0.05	0.23	0	0
**Approach/escape**																
Head alignment	23.84	13.32	26.45	15.17	31.74	16.97	26.12	17.03	19.76	13.66	22.23	14.83	27.81	15.08	25.84	16.46
Nose/hand stick	1.72	4.66	1.28	3.95	0.72	2.17	1.52	3.71	1.27	2.55	0.67	1.99	0.48	1.51	1.04	3.08
Reject. the stick	0.25	0.55	0.20	0.52	0.21	0.42	0.10	0.31	0.40	0.68	0.40	0.68	0.21	0.54	0.20	0.41

A verification of the results using a Friedman test, which is a more conservative test allowing more than two conditions to be compared, and thus enabling the previous comparison to be extended to the control modality, confirms the significant influence of the hedonic modality on two indicators. The results confirm the significant effect of the hedonic modality on the duration of participants’ head alignment on the stick [*X*^2^ (2, *n* = 20) = 6.30, *p* = 0.021], with an effect size that could be described as small (*W* = 0.178) and pairwise comparisons with Dunn-Bonferroni corrected for multiple testing showing that the head alignment is significantly longer when participants are exposed to pleasant compared to unpleasant odors (*p* = 0.026). The comparison of the three modalities (un/pleasant and control) also showed a significant effect of the hedonic modality on pouting [*X*^2^ (2, *n* = 20) = 18.29, *p* = 0.000], with an effect size that could be described as moderate to large (*W* = 0.457) and pairwise comparisons with Dunn-Bonferroni correction revealing that the participants exhibited significantly more pouting when faced with unpleasant odors compared to pleasant (*p* = 0.002), as well as when they faced unpleasant odors compared to control (*p* = 0.002).

### 3.2. Participants’ responses during the first exposure to odors vs. the second

The second analysis aimed to study whether the differences in hedonic responses were already present during the first exposure to the odor (T1) in comparison to the second (T2). The analyses were performed on all participants (*n* = 20). The results show that differentiated responses were already present during the first exposure. At the first exposure, participants vocalized positively significantly longer in the presence of pleasant odors (*M*_Pleasant_ = 0.53 s, SD = 1.67, *M*_Unpleasant_ = 0.13 s, SD = 0.35, *Z* = −2.56, *p* = 0.006) and made more pouts in the presence of unpleasant ones (*M*_Unpleasant_ = 0.76, SD = 1.12, *M*_Pleasant_ = 0.33, SD = 0.96), *Z* = −3.41, *p* < 0.001, with mean effect sizes (*r* = 0.20 and *r* = 0.27 respectively). With a smaller effect size (*r* = 0.14), participants smiled significantly more in the presence of pleasant odors than in the presence of unpleasant odors (*M*_Pleasant_ = 2.19 s, SD = 4.17, *M*_Unpleasant_ = 1.43 s, SD = 3.05, *Z* = −1.72, *p* = 0.043).

At the second exposure, participants vocalized positively for significantly longer in the presence of pleasant odors (*M*_Pleasant_ = 0.59 s, SD = 2.19, *M*_Unpleasant_ = 0.27 s, SD = 1.42, *Z* = −2.39, *p* = 0.009, *r* = 0.19). In contrast, they made significantly more pouting and faces (*M*_Unpleasant_ = 0.95, SD = 1.54, *M*_Pleasant_ = 0.33, SD = 0.97, *Z* = −3.89*, p* < 0.001) and longer negative vocalizations (*M*_Unpleasant_ = 0.195 s, SD = 0.71, *M*_Pleasant_ = 0.12, SD = 0.86, *Z* = −2.04, *p* = 0.021), in the presence of unpleasant stimuli, with moderate (*r* = 0.31) and small (*r* = 0.16) effect sizes, respectively. They exhibited nausea reactions only with unpleasant odorants (*M*_Unpleasant_ = 0.08, SD = 0.31, *M*_Pleasant_ = 0, SD = 0, *Z* = −2.12, *p* = 0.034, *r* = 17). With smaller effect sizes, participants held their heads aligned on the stick significantly longer (*M*_Pleasant_ = 14.02 s, SD = 9.62, *M*_Unpleasant_ = 11.92 s, *SD* = 8.34, Z = −1.94, *p* = 0.026, *r* = 0.15) and smiled more in the presence of pleasant odors (*M*_Pleasant_ = 2.14 s, SD = 4.29, *M*_Unpleasant_ = 1.48 s, SD = 3.75, *Z* = −1.65, *p* = 0.049, *r* = 0.13).

Emotional reactions at Time 1 and Time 2 considered separately are consistent with the previous analysis on T1 and T2 aggregated, with participants showing, on average, more positive emotional reactions when presented with pleasant odors and more negative emotional reactions when presented with unpleasant odors. However, we note only 3 significant indicators out of 9, at T1, but 6 out of 9, at T2. Table 5 shows the significant emotional reactions in each of the odorant presentation times.

**Table 5:** Summary of the significant results for the different measurement times.

Responses	Sign. asymp *p*/effect size *r*		
	T1 *N* = 20^a^	T2 *N* = 20^a^	T1 + T2 *N* = 20^a^
**Positive**			
Smiling	*/0.14	*/0.13	*/0.14
Positive vocalizations	**/0.20	**/0.19	**/0.25
**Negative**			
Emotional outburst			
Pouting, making a face	**/0.27	***/0.31	***/0.37
Negative vocalizations		*/0.16	
**Physiological reaction**			
Nausea reactions		*/0.17	*/0.17
**Approach/escape behavior**			
Head alignment on the stick		*/0.15	*/0.17
Nose/hand on the stick			
Rejecting the stick			

*p < 0.05, **p < 0.01, ***p < 0.001, Pleasant odors > Unpleasant odors; Pleasant odors < Unpleasant odors.

a 19 participants completed 4 sessions while one participant completed only 3 sessions.

### 3.3. Impact of the mood on the participants’ olfactory hedonic responses

The purpose of this analysis was to answer the research question about the influence of the participants’ general mood on their behavioral responses to odors. For this analysis, the participants were divided into two groups based on their MIPQ scores. The first group includes seven participants considered to have a low general mood (score total < 60); the other 13 participants were considered to have a high general mood (score total > 60). The mean overall MIPQ score is *M* = 46.12 (SD = 6.64) for the low mood group and *M* = 74.54 (SD = 6.57) for the high mood group. A generalized linear mixed model analysis ( α=0.05) was performed with group membership (high vs. low mood), the hedonic value of the stimulus (pleasant vs. unpleasant) and their interaction as independent variables.

**Figure 2:**
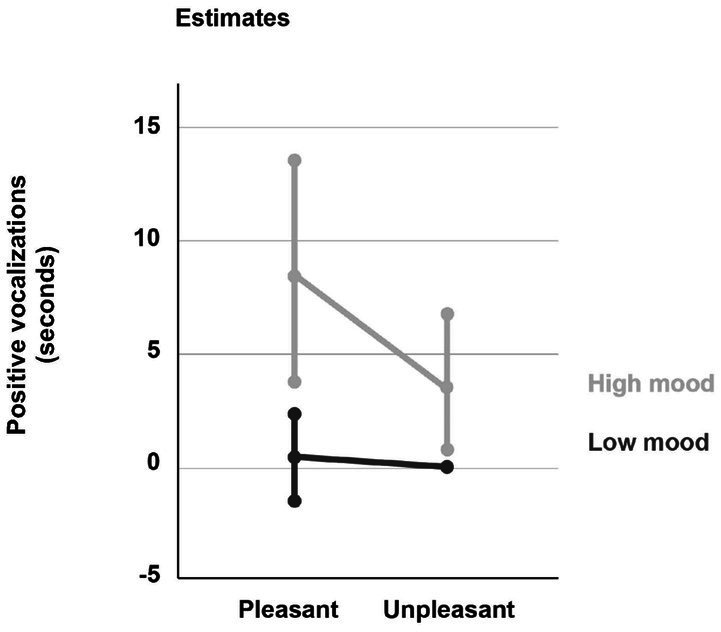
Effect of mood and odor pleasantness on average (±standard deviation) duration of positive vocalizations.

The results show an interaction effect of mood and hedonic value of the stimuli on positive vocalizations. On average, participants vocalized positively longer when presented with pleasant odors (*M* = 0.47 s, SD = 0.14) than when presented with unpleasant odors [*M* = 0.18 s, SD = 0.07, *F*(1, 154) = 8.56, *p* = 0.004]. In addition, participants with a high mood vocalized longer (*M* = 0.62 s, SD = 0.19) than those with a low mood [*M* = 0.03 s, SD = 0.03, *F*(1,154) = 8.82, *p* = 0.003]. The significant interaction effect between the two factors indicates that the response of participants with high mood to pleasant vs. unpleasant odors differs significantly from those of participants with low mood (see Figure 2).

About simple interactions, the results show a significant main effect of hedonic valence of odors on the head alignment duration on the stimulus [*F*(1, 154) = 11.19, *p* = 0.001] with participants remaining aligned significantly longer on pleasant (*M* = 28.53, SD = 2.2) than on unpleasant odorants (*M* = 25.39, SD = 2.26). This result is consistent with that of the first analysis. A second main effect was noted for this indicator [*F*(1, 154) = 8.01, *p* = 0.005], participants with low mood aligning their head on the stick for significantly longer (*M* = 33.13, SD = 3.91) than participants with high mood (*M* = 20.79, SD = 1.93). The third effect is that of participants’ mood on smile duration [*F*(1, 154) = 6.69, *p* = 0.011], participants with high mood smiling significantly longer (*M* = 4.86, SD = 1.51) than those with low mood (*M* = 0.76, SD = 0.47). With respect to positive vocalizations, two main effects were found: one with mood [*F*(1, 154) = 8.82, *p* = 0.003], the other with the hedonic valence of odors [*F*(1, 154) = 8.56, *p* = 0.004]. Participants with high mood vocalized positively significantly longer (*M* = 0.62, SD = 0.19) than those with low mood (*M* = 0.03, SD = 0.03), and pleasant odors triggered significantly longer positive vocalizations (*M* = 0.47, SD = 0.14) compared to unpleasant (*M* = 0.18, SD = 0.07). Finally, a significant main effect was found with respect to negative vocalization duration and mood [*F*(1, 154) = 4.84, *p* = 0.029], with participants with high mood vocalizing negatively significantly longer (*M* = 0.23 s, SD = 0.10) than those with low mood (*M* = 0.001, SD = 0.001).

### 3.4. Impact of the age and the feeding mode on the participants’ olfactory hedonic responses

No interactions effect were found between the hedonic value of the stimuli and the participants’ age, or their feeding mode, on the behavioral responses to odors.

## 4. Discussion

To date, the abilities of individuals with PIMD to express emotions have mainly been extrapolated from reporting by proxies (11). This research offers a systematic analysis of the responses of participants with PIMD to pleasant and unpleasant odorants. Three results stand out: first, the participants expressed hedonically differentiated and congruent responses to odorous stimuli of contrasted valence; second, their expressive and vocal repertoires toward odor-borne hedonic modalities seem basically the same as those observed in typical pre-verbal populations; third, the results show that their general mood has a significant influence on their emotional reactions.

Emotions are adaptive and serve as a basis for reacting to contextual events and forming representations. They also enable organisms to signal what contributes to their well-being (53). In this sense, confirming the existence of emotional discrimination abilities in individuals with PIMD is an important step and suggests a first lever for understanding their emotional balance. To our knowledge, it is the first time that an interaction effect of both mood and stimuli valence is found in this population. In a study involving 27 participants with severe and profound intellectual disability, Vos et al. (54) found single effects between stimuli valence, mood and participants’ behavioral emotion score, but they did not find any links between the three parameters. In our research, participants with low mood expressed significantly fewer contrasted emotional reactions both to pleasant and unpleasant odors, in comparison to peers with higher mood. Three models studied the link between mood and emotions (54): the Positive Attenuation model suggests that depressed people show attenuated physiological responses to stimuli with a positive valence, but that they do not differ from nondepressed individuals in their reaction to unpleasant, negatively valenced, stimuli (55); the Negative Potentiation hypothesis states that depression increases reactivity to negative stimuli that are felt even more negatively in comparison to nondepressed people, positive stimuli reactivity remaining preserved (56); finally the Emotion Context Insensitivity theory (57), with which our results are best aligned, states that low mood, depression risk, and especially diagnosed depression, lead to a general disengagement with the environment and a lack of contextually appropriate reactions to both *positive and negative* stimuli. Of course, emotion reactivity and emotional behaviors are two different sides of the same coin, but the comparison with these theories may draw attention to the influence of mood in the responses of people with PIMD to hedonic stimuli. It also highlights the importance of assessing emotional multiaxial indices across these individuals’ development and contexts, as for typical ones (58). Finally, our findings raise the question of the gradation of the emotions expressed, as pleasant odors trigger both significant emotional and appetitive responses, and unpleasant only emotional ones. This result can be interpreted in different ways, either people with PIMD only have limited experience of opportunities to disengage, which seems to be contradicted by the statistically significative presence of rejection reactions to odorous stimuli of any valence vs. controls; or unpleasant stimuli are sufficient to elicit negative hedonic facial reaction, but not gestural responses, which might suggest a gradation of reactions. A greater variety of responses, which was not part of the research questions, was observed during the second stimulus exposure. With regard to this result, two points come to mind. Either the repetition of the stimulus presentation offers additional time for odor exploration and helps the participants to confirm and enrich their first impressions and reactions. Or their responses are functionally correlated, and graded in terms of intensity of response between first and second presentation. From an applied perspective, and in terms of information processing and pedagogical implications, a confirmation of these interpretations might clarify the fact that the hedonic response is not fully expressed during the first contact with the stimulus and that a second exposure is necessary to trigger a fuller and more marked expression of preferences.

### 4.1. Limitations

Despite various precautions, this study has several limitations. The perceptual properties that is intensity, familiarity, and the trigeminal properties, were only partially or not controlled at all while this may play a salient role on odor perception and especially valence attribution (59). A second limitation is that only one of the three coders was fully blind to the experimental conditions. Another weakness is that the sample, which was heterogeneous in terms of disabilities and syndromes, can question the integrity of the participants’ olfactory system. First because, as in the typical population, they are exposed to acquired factors, such as sinonasal and upper respiratory tract infections that can alter olfaction (60). Second, because individuals with developmental delay or epilepsy are overrepresented in pediatric population with radiological anomalies of the olfactory system (61). Although the literature did not report any relationship between the participants’ disabilities and syndromes and olfaction, olfactory impairment has been reported in mesial temporal lobe epilepsy due to unilateral hippocampal sclerosis (62), and in prenatal hypoxia or herpes simplex virus in rare animal research (63, 64). It is also well known that medication can modify olfactory perception and people with PIMD are heavy consumers of medication. In our study, a medical student and a professor of pharmacology helped us to analyze the participants’ medications and to calculate the percentage of those known to interfere with the sense of smell. The analysis was based on the literature as well as the monographs about Swissmedic-approved drugs.^2^ Thirty-six active constituents (see Supplement 1) were identified corresponding to nearly 50 brand names, among which four were known for a potential side effect on olfaction, i.e., Topiramate (an antiepileptic), Risperidone (an antipsychotic), morphine, and Levothyroxine (hormones). Two participants were taking Topiramate, two Risperidone, one morphine and one hormonal medication; we cannot rule out that side effects on olfaction could have occurred in one or more of them. The wide participants’ age range may explain why no age effect on olfactory preferences was found, in contrasts to the typical population (22). A control group would have been welcome. Finally, asking typical children, and not typical adults, to characterize the intensity as well as the hedonic dimension of the stimuli would have made it possible to take better account of the developmental factor.

## 5. Conclusion and perspectives

In spite of these limitations, this study provides a more in-depth knowledge of the olfactory hedonic functioning of children and adolescents with PIMD. It also shows that despite the severity of the participants’ impairment, odors trigger clearly contrasted emotions in most participants, emphasizing the relevance of using olfactory simulation for cognitive, emotional, regulation, and well-being purposes. In this approach, given their strong interconnections, the use of odors to support emotional expression and mood regulation opens particularly interesting intervention perspectives, including the use of pleasant potentially comforting odors for therapeutic interventions. The findings should invite caregivers to pay systematized attention to somatic and whole-body responses, i.e., nauseous reactions, in daily and research settings when people are nonverbal or have few means of action on the world. Alternatives have to be found to substances inducing aversive reactions, e.g., the hand sanitizer that is used daily by educational personnel working with the participants. Finally, the findings remind us that emotional competence supposes access to the full range of emotional experiences, and that the presence of negative emotions, because of their adaptive function, is not worrying in itself, contrary to a blunted or overly vivid emotional expression (65).

## Data availability statement

The consolidated data supporting the conclusions of this article is available on request.

## Ethics statement

The studies involving human participants were reviewed and approved by Geneva and Vaud ethics committees (ID: 2019-0023 4), which are part of the national organisation Swissethics. Written informed consent to participate in this study was provided by the participants' legal guardian/next of kin.

## Author contributions

GP and JD: concept and design. CB, JD, and GP: acquisition, analysis, and interpretation of data. GP and CB: drafting of the manuscript. GP: critical revision of the manuscript. CB and GP: statistical analysis. All authors contributed to the article and approved the submitted version.

## Funding

This work was supported by Givaudan Foundation, Stiftung “Heilpädagogisches Zentrum” (Special Education Foundation), and University of Fribourg.

## Conflict of interest

The authors declare that the research was conducted in the absence of any commercial or financial relationships that could be construed as a potential conflict of interest.

## Publisher’s note

All claims expressed in this article are solely those of the authors and do not necessarily represent those of their affiliated organizations, or those of the publisher, the editors and the reviewers. Any product that may be evaluated in this article, or claim that may be made by its manufacturer, is not guaranteed or endorsed by the publisher.

## Footnotes

^1^ www.burghart-mt.de

^2^ https://www.swissmedicinfo.ch/?Lang=EN
