# Supplementary material for: Odor hedonic responses in children and young people with profound intellectual and multiple disabilities
Source: Front Psychiatry. 2023 Aug 24;14:1066286. doi: 10.3389/fpsyt.2023.1066286 (PMC10484511; doi:10.3389/fpsyt.2023.1066286)
Supplement: Supplementary file 1 [file Table_1.docx]

**Supplement 1**

Adapted from Leite Costa, G. (2021). Polyhandicap et médication: les médicaments ont-ils une influence sur l’olfaction? [Profound intellectual and multiple disabilities and medication: do drugs have an influence on olfaction?]. Master's thesis submitted to the Faculty of Medicine of the University of Fribourg (Switzerland). Supervisors: Prof. G. Petitpierre and Dr J. Vukovic.

**Table 1.** Active constituents consumed by the participants of the hedonic sub-study. Occurrences per drug classes and potential side-effects on olfactory and/or taste functioning^1^

| Drug classes, occurrences | Active constituents, N=36 | Potential side-effect on olfactory functioning^2^ | Potential side-effect on taste functioning^2^ |
| --- | --- | --- | --- |
| Antiepileptics, n=7 | Acide valproïque |  |  |
|  | Carbamazépine |  | Yes |
|  | Oxcarbazépine |  |  |
|  | Lamotrigine |  |  |
|  | Lévétiracétam |  |  |
|  | Rufinamid |  |  |
|  | Topiramate | Yes | Yes |
| Benzodiazepines, n=2 | Clonazépam |  | Yes |
|  | Clobazam |  |  |
| Muscle relaxants, n=2 | Baclofène |  | Yes |
|  | Tizanidine |  |  |
| Antidyskinesics, n=1 | Tétrabénazine |  |  |
| Antipsychotics, n=3 | Olanzapine (atypical) |  |  |
|  | Rispéridone (atypical) | Yes | Yes (metallic taste) |
|  | Chlorpromazine (typical) |  |  |
| Hypnotiques-sédatifs, n=2 | Hydrate de chloral |  |  |
|  | Mélatonine |  |  |
| Proton pump inhibitors (PP), n=2 | Oméprazole |  | Yes |
|  | Ésoméprazole |  | Yes |
| Antiulcerants, n=1 | Ranitidine |  | Yes |
| Antacids, n=1 | Magaldrate |  |  |
| Antiflatulents, n=1 | Siméticone |  |  |
| Laxatives, n=3 | Lactulose (osmotic) |  |  |
|  | Macrogol (osmotic) |  |  |
|  | Paraffine (lubricant) |  |  |
| Vitamins and minerals, n=3 | Cholécalciférol |  | Yes (metallic taste) |
|  | Cholécalciférol et calcium |  | Yes (metallic taste) |
|  | Zinc | Yes (intranasal only) | Yes (metallic taste) |
| Others, n=2 | Lactibiane® |  |  |
|  | Fantomalt® |  |  |
| Analgesics, n=2 | Paracétamol |  | Yes |
|  | Morphine | Yes | Yes |
| Corticosteroids, n=1 | Hydrocortisone | Yes (intranasal only) | Yes |
| Hormones, n=2 | Lévothyroxine | Yes | Yes |
|  | Somatropine |  |  |
| Contraceptives h., n=1 | Médroxyprogestérone |  |  |

**Legend:** ^1^According to Ackerman & Kasbekar (1997); Doty & Bromley (2004); Henkin (1994); Lötsch et al. (2012); Lötsch et al. (2015); Schiffman (2018) extracted after a search in Pubmed (Keywords: drug AND taste OR smell OR olfaction AND Profound intellectual and multiple disabilities). ^2^As a reminder, the absence of content in these columns indicates that information on olfactory/taste impairment is neither described in the quoted literature, nor in Swissmedicinfo.

**Bibliography**

Ackerman BH, and Kasbekar N. Disturbances of taste and smell induced by drugs. *Pharmacotherapy*. (1997) 17:482–96.

Doty RL, and Bromley SM. Effects of drugs on olfaction and taste. *Otolaryngol Clin North Am.* (2004) 37:1229–54. <https://doi.org/10.1016/j.otc.2004.05.002>

Henkin RI. Drug-induced taste and smell disorders. Incidence, mechanisms, and management related primarily to treatment of sensory receptor dysfunction. *Drug Saf*. (1994) 11:318–77. <https://doi.org/10.2165/00002018-199411050-00004>

Lötsch J, Geisslinger G, and Hummel T. Sniffing out pharmacology: interactions of drugs with human olfaction. *Trends Pharmacol. Sci*. (2012) 33:193–199. <https://doi.org/10.1016/j.tips.2012.01.004>

Lötsch J, Knothe C, Lippmann C, Ultsch A, Hummel T, and Walter C. Olfactory drug effects approached from human-derived data. *Drug Discov. Today*. (2015) 20:1398–406. <https://doi.org/10.1016/j.drudis.2015.06.012>

Schiffman SS. Influence of medications on taste and smell. *World J. Otorhinolaryngol. Head Neck Surg*. (2018) 4:84–91. <https://doi.org/10.1016/j.wjorl.2018.02.005>
